# Somatostatin Analogues for Receptor Targeted Photodynamic Therapy

**DOI:** 10.1371/journal.pone.0104448

**Published:** 2014-08-11

**Authors:** Slávka Kaščáková, Leo J. Hofland, Henriette S. De Bruijn, Yunpeng Ye, Samuel Achilefu, Katy van der Wansem, Angelique van der Ploeg-van den Heuvel, Peter M. van Koetsveld, Michael P. Brugts, Aart-Jan van der Lelij, Henricus J. C. M. Sterenborg, Timo L. M. ten Hagen, Dominic J. Robinson, Martin P. van Hagen

**Affiliations:** 1 Center for Optical Diagnostics and Therapy, Department of Radiation Oncology, Erasmus MC, Rotterdam, The Netherlands; 2 Department of Internal Medicine, Erasmus MC, Rotterdam, The Netherlands; 3 Department of Otolaryngology and Head & Neck Surgery, Erasmus MC, Rotterdam, The Netherlands; 4 Department of Radiology, School of Medicine, Washington University, St. Louis, Missouri, United States of America; 5 Department of Immunology, Erasmus Medical Center, Rotterdam, The Netherlands; 6 Department of Surgical Oncology, Erasmus Medical Center, Rotterdam, The Netherlands; MGH, MMS, United States of America

## Abstract

Photodynamic therapy (PDT) is an established treatment modality, used mainly for anticancer therapy that relies on the interaction of photosensitizer, light and oxygen. For the treatment of pathologies in certain anatomical sites, improved targeting of the photosensitizer is necessary to prevent damage to healthy tissue. We report on a novel dual approach of targeted PDT (vascular and cellular targeting) utilizing the expression of neuropeptide somatostatin receptor (sst_2_) on tumor and neovascular-endothelial cells. We synthesized two conjugates containing the somatostatin analogue [Tyr3]-octreotate and Chlorin e6 (Ce6): Ce6-K_3_-[Tyr3]-octreotate (**1**) and Ce6-[Tyr3]-octreotate-K_3_-[Tyr3]-octreotate (**2**). Investigation of the uptake and photodynamic activity of conjugates *in-vitro* in human erythroleukemic K562 cells showed that conjugation of [Tyr3]-octreotate with Ce6 in conjugate **1** enhances uptake (by a factor 2) in cells over-expressing sst_2_ compared to wild-type cells. Co-treatment with excess free Octreotide abrogated the phototoxicity of conjugate **1** indicative of a specific sst_2_-mediated effect. In contrast conjugate **2** showed no receptor-mediated effect due to its high hydrophobicity. When compared with un-conjugated Ce6, the PDT activity of conjugate **1** was lower. However, it showed higher photostability which may compensate for its lower phototoxicity. Intra-vital fluorescence pharmacokinetic studies of conjugate **1** in rat skin-fold observation chambers transplanted with sst_2_^+^ AR42J acinar pancreas tumors showed significantly different uptake profiles compared to free Ce6. Co-treatment with free Octreotide significantly reduced conjugate uptake in tumor tissue (by a factor 4) as well as in the chamber neo-vasculature. These results show that conjugate **1** might have potential as an *in*-vivo sst_2_ targeting photosensitizer conjugate.

## Introduction

In PDT visible light is used to transfer energy to a photosensitizer. This leads to the production of cytotoxic intermediates, such as singlet oxygen or free radicals that result in cell death and tissue response [1]. With the use of fiber optic devices and endoscopy, light can be delivered to almost any part of the body, significantly increasing the applications of PDT. For the ablation of deep-seated solid tumors, interstitial approaches are used [2]. PDT is approved as a treatment for neovascular age related macular degeneration and (pre-) cancerous conditions such as superficial gastric cancer, Barrett's esophagus, the palliative treatment of head and neck cancers, and skin malignancies [3].

The ability to confine photosensitizer activation by restricting illumination to the tumor allows a certain degree of selectivity. However, for applications of PDT in complex anatomical sites, such as the abdominal or thoracic cavities, this is not possible and in circumstances where the selectivity of traditional photosensitizers is insufficient, targeted photosensitizer delivery becomes essential. A targeted approach employs the utilization of ligands, which can bind specifically to neovascular endothelium or cellular markers to target tumor tissue. While antibody-conjugates have received the most attention [4]–[7], cellular transformations offer other potent targets to exploit. Growth factor receptors, hormonal-, low-density lipoprotein-, transferrin, glucose, folic acid-, and insulin- receptors have been investigated as cellular markers for active photodynamic targeting [8]–[10]. In addition to the overall aim of increasing the uptake of photosensitizers in target cells, the small radius of action of singlet oxygen (0.1–0.2 µm) [11] has lead investigators to deliver conjugates to specific sites within cells. Targeting of nuclear receptors is one such example [10].

Extensive research in targeted PDT has shown that there are several factors that affect the efficiency of *in vivo* tumor targeting. The chemical modifications that are necessary for the synthesis (conjugation of photosensitizer with ligand) may lead to a reduced affinity of the ligand to its receptor or to a reduced singlet oxygen quantum yield. In an ideal scenario the photosensitizer and ligand affinity will be preserved, the receptor would be present on the surface of tumor cells at high concentrations compared to normal cells and lead to an internalization process that enhances PDT efficacy [9].

Based on the development of targeted PDT, the somatostatin receptor (sst) represents an attractive option for tumor targeting. Ssts are over-expressed in a large number of human cancers. Receptor density is high and the distribution is usually homogeneous [12]. Among the 5 sst subtypes, the sst_2_ predominates [12], [13]. This receptor represents the basis for a number of clinical applications, e.g. symptomatic therapy with somatostatin analogues. Moreover, for diagnostic purposes, sst scintigraphy is an important tool for a subgroup of neuroendocrine tumors (NET) [14]. Radiotherapy with radiolabeled somatostatin analogues is also extremely promising for patients with gastroenteropancreatic sst_2_-positive NET [15], [16]. Previous studies have shown that angiogenic vessels, as well as peri-tumoral vessels express ssts, which is predominantly sst_2_ [17]–[20]. It is accepted that somatostatin acts locally on tumor growth, either through direct action on tumor cells and/or through action on peritumoral vessels [21]. Upregulated ssts in tumors makes the sst an attractive cellular target, since a photosensitizer-conjugate can be used to target tumor cells as well as neovasculature. Our aim was to develop an approach to improve PDT by direct conjugation of photosensitizer with the synthetic, metabolically stable somatostatin analogue octreotate.

We report on the synthesis and *in vitro* cytotoxicity of two Ce6-somatostatin analogue conjugates: Ce6-K_3_-[Tyr3]-octreotate (**1**) and Ce6-[Tyr3]-octreotate-K_3_-[Tyr3]-octreotate (**2**). Their *in vitro* quantitative uptake and phototoxicity in sst_2_^+^ K562 and WT cells have been compared with those of un-conjugated Ce6. Intra-vital *in-vivo* fluorescence pharmacokinetics of conjugate **1** was determined in rat skin-fold observation chambers transplanted with sst_2_^+^ AR42J pancreatic tumor cells.

## Materials and Methods

In a series of studies on photosensitizer conjugation with octreotate, we began with the development of 2-[1-Hexyloxyethyl]-2-devinyl pyropheophorbide-a (HPPH)-octreotate conjugates. We selected HPPH because it is considered as a potent and promising clinical photosensitizer [22]. However, our preliminary *in vitro* studies revealed that the lipophilicity of HPPH dominated the specific recognition of the conjugate by the sst_2_ receptor and led to non-specific uptake (unpublished data). We hypothesized that coupling photosensitizers that are more hydrophilic would be a more suitable approach. To test this hypothesis, Chlorin e6 (Ce6) was selected as a photosensitizer. Ce6 is characterized by rapid elimination from the body [23]–[26], high absorption in the red spectral region and high sensitizing efficacy [26]. Although, Ce6 is very photolabile [27], [28], its high solubility in physiological media [23], [24] overcomes the delivery problems of hydrophobic photosensitizers.

**Figure 1:**
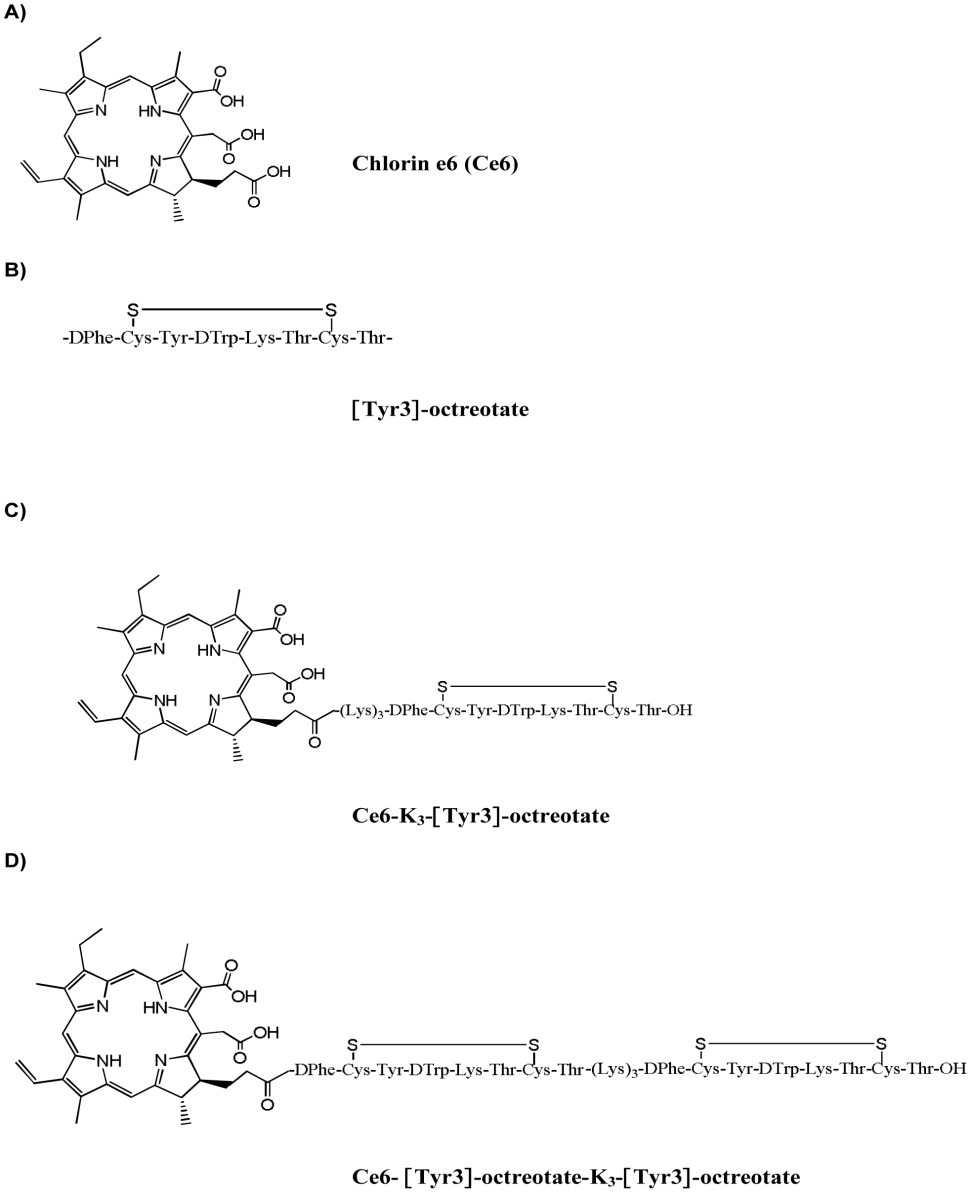
Structures of Ce6 and its two [Tyr3]-octreotate conjugates. (A) Ce6; (B) [Tyr3]-octreotate motif; (C) Ce6-K_3_-[Tyr3]-octreotate (conjugate **1**) and (D) Ce6-[Tyr3]-octreotate-K_3_-[Tyr3]-octreotate (conjugate **2**). A tri-lysine linker (K_3_) was used between Ce6 and [Tyr3]-octreotate motifs to improve the hydrophilicity of the monomeric conjugate **1**. A similar linker was also inserted between the two [Tyr3]-octreotate motifs in the dimeric analog, conjugate **2**.

The structures of Ce6 and 2 somatostatin analogue conjugates of Ce6, used in the present study are shown in Figure 1.

### Synthesis of Ce6-coupled somatostatin analogues and characterization (File S1)

The synthesis of Ce-6-coupled somatostatin analogues is summarized in Schemes S1 and S2 in File S1. Scheme S1 shows the synthesis of Ce_6_-K_3_-[Tyr3]-octreotate (conjugate **1**), whereas Scheme S2 summarizes the synthesis of Ce_6_-[Tyr3]-octreotate-K_3_-[Tyr3]-octreotate (conjugate **2**). The Figure S1 in File S1 shows the ES-MS and HPLC profiles of conjugates **1** and **2**.

### Cells Transfection and Culture

**Figure 2:**
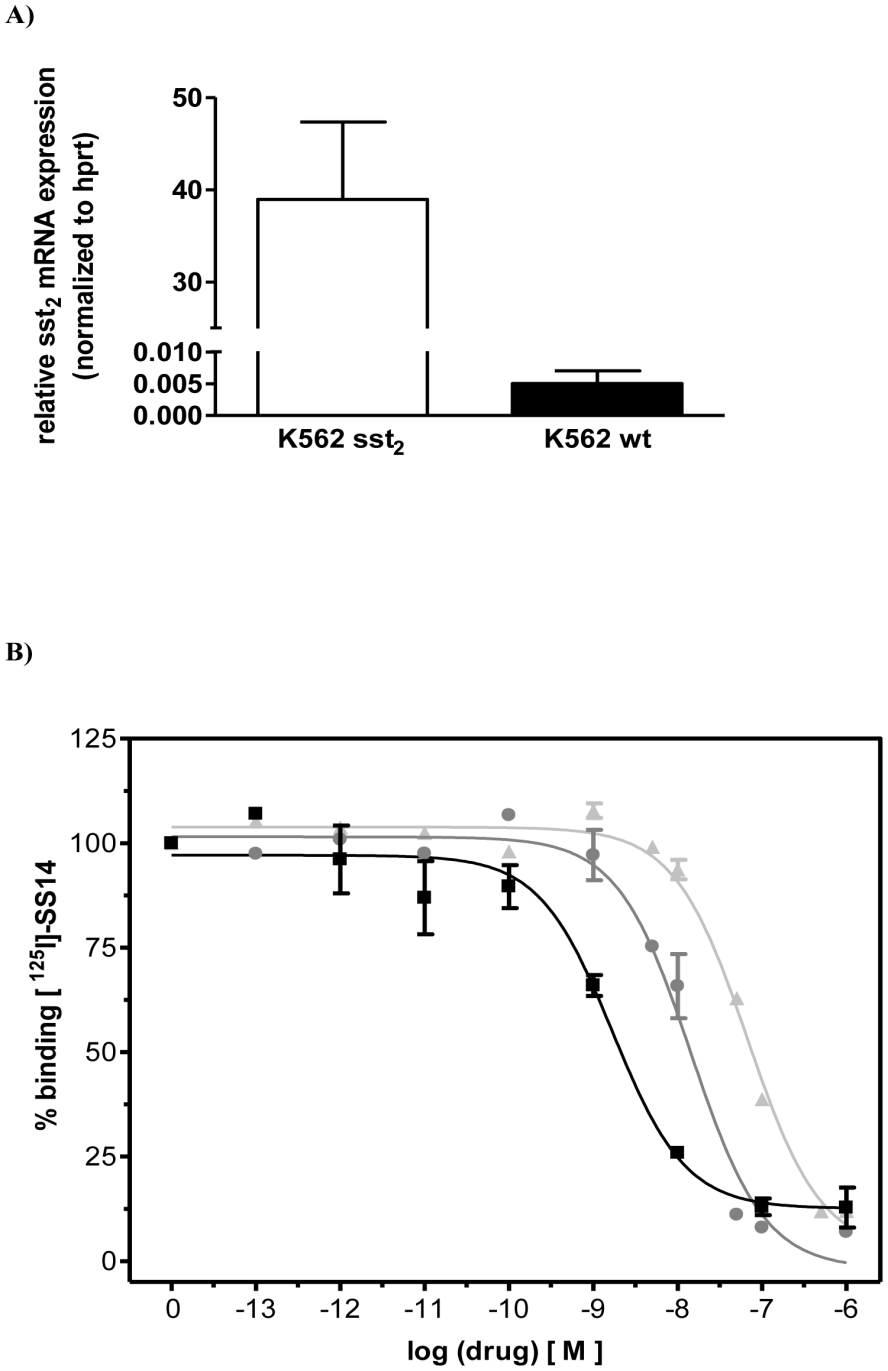
(A) Comparison of sst_2_ mRNA expression in transfected human myeloid K 562 sst_2_^+^ and WT cells; (B) Displacement of [^125^I]-SS14 binding to membrane preparations of K562 sst_2_^+^ cells, by unlabeled Octreotide (▪), and by conjugates 1 (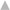) and 2 (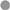).

Copy DNA of the human sst_2_ receptor was merged into a LZRS-lyt2-IRES plasmid background (lyt2 being the mouse CD8 receptor). The calcium precipitation method [29] was used to transfect non-adherent K562 cells with the LZRS-lyt2-IRES-sst_2_. Expression of lyt2 was evaluated by means of FACS analysis using FITC labeled anti-mouse CD8 antibody (BD PharMingen, Netherlands), 1∶160 diluted in FACS-buffer. Clones were grown form Lyt2 positive isolated cells. Vital clones were cultured and tested in time for stability of sst_2_ receptor expression, by means of both FACS analysis (using FITC labeled octreotate 200 nM) as well as sst_2_ mRNA expression by RT-PCR as previously described [30]. Clone showing high and stable somatostatin receptor expression (Fig. 2A) was used in further experiments. K562 cells were grown in RPMI 1640 containing L-glutamine, 10% heat-inactivated FCS, 50 µg/ml penicillin and 50 µg/ml streptomycin, and were maintained in an incubator at 37°C in an humidified atmosphere of 5% CO_2_.

### Photophysical properties of the conjugates (File S1)

The partition coefficient, steady-state fluorescence and absorption properties, photobleaching properties, singlet oxygen quantum yield and the pH properties of the conjugates were determined. The details about used methods are part of File S1 and as described previously [31]–[37].

### Cellular uptake

Stock solutions of Ce6 and Ce6-conjugates (1 mM) were dissolved in 80% DMSO and were stored in the dark at −20°C. Before experiments, the solutions were diluted to a concentration of 0.1 mM in 20% DMSO. K562 sst_2_+ or WT (3×10^5^) in 1 ml RPMI 1640 serum-free or supplemented with 5% FCS were incubated with Ce6 or with conjugate (1 µM) for 15 min to 24 h in the dark at 37°C. After incubation, the cells were centrifuged, cell pellet dissolved in 2% SDS and fluorescence emission spectra (λ_exc_ = 405 nm) recorded. Photosensitizer concentrations were calculated in triplicate according to a calibration curve generated by mixing a known concentration of photosensitizer with a lysated control samples.

### Membrane binding studies with [^125^I]-SS14

The binding potency of Ce6-conjugates to sst_2_ on K562 sst_2_^+^ cells was evaluated on cell membrane preparations. The quantification of conjugates binding was determined by competition binding assay (displacement of [^125^I]-SS14 binding from sst by increasing concentrations of unlabeled Octreotide or conjugates) as described previously [30].

### Confocal Microscopy

Confocal fluorescence imaging (LSM 510 Meta (Zeiss, Germany)) was performed using a Plan-apochromat 63×/1.4 oil objective. The subcellular distribution of photosensitizer fluorescence was mapped under 405 nm diode laser excitation in the lambda mode (539–753 nm).

### Image Analysis

The spectra were analyzed as a linear combination of basis spectra and fitted using a singular value decomposition algorithm. The image analysis was performed using the Labview 7.1 (National Instruments Corporation). Basis spectra of components and cell autofluorescence were measured by the same microscopic system. The images were reconstructed in RGB format: blue represents autofluorescence and green photosensitizer-fluorescence.

### Phototoxicity

*In vitro* PDT experiments were performed to determine the phototoxicity of free Ce6 and conjugates. K562 sst_2_+ and WT cells were seeded in L-polylysine-coated (10 µg/ml) 96-well (8×10^3^/well) and 24-well (4×10^4^/well) and incubated overnight in 5% CO_2_ at 37°C. After 24 hours, cells were treated for 4 h with photosensitizer (0.01–1 µM) in the absence or presence of excess of unconjugated Octreotide (10^−5^ M) in serum-free medium. After incubation, cells were washed. Fresh serum-free medium was added, and cells were illuminated with 652 nm to a dose from 0.5 to 5 J cm^−2^ at 5 mW.cm^−2^. Thereafter, serum-containing medium was added and cells were allowed to grow for an additional 3 days in 5% FCS medium. Toxicity was determined by: measurement of DNA contents using the bisbenzimide fluorescent dye (Hoechst 33258, Boehring Diagnostics, CA, USA) and measurement of mitochondrial dehydrogenase cleavage of WST-1 reagent (Roche Diagnostics, Germany) [38], [39]. Experiments were done in triplicates.

### *In-vivo* pharmacokinetics in AR42J tumours in the rat skin-fold chambers

Intra-vital confocal fluorescence imaging was performed in single sided rat skin-fold chambers transplanted with sst_2_^+^ AR42J acinar pancreas tumors as described previously [40]. The study was approved by the animal experimental committee of the Erasmus MC. Chambers were prepared in 12 animals divided into three groups. Animals were anaesthetized (ketamine (100 mg/kg) i.p. and xylazine (10 mg/kg) s.c. and AR42J cells (1–3 10^5^ cells) were inoculated superficially in the facia/sub-cutaneous tissue and an 18 mm round cover slide was placed and fixed to close the window. On the surrounding tissue Bactroban was applied to prevent inflammation and analgesia (Rimadyl Cattle 5 mg/kg) was administered subcutaneously. Pharmacokinetic experiments were initiated when tumors were clearly visible and had a microscopically adequate vascular supply. Solutions of free Ce6 and Conjugate **1** were made by dissolving in 80% DMSO and diluting with PBS to a final concentration of 5% DMSO. The first group of animals received Ce6 at a dose of 0.2 mg/kg, the second group received conjugate **1** at a dose of 0.67 mg/kg such that the same number of Ce6 molecules were administered to each animal; (equivalent Ce6 concentration of 2.011×10^−4^ M). A third group of animals was pre-administered with free Octreotide, at a dose of 0.5 mg/kg.

### Intra-vitial confocal microscopy, image analysis and statistics

Animals were anesthetized using isoflurane/O_2_ and autofluorescence images were recorded. Drug was administered intravenously into the tail vein. Fluorescence images were acquired 5 and 20 minutes after conjugate administration after which the animal was allowed to recover. Subsequent fluorescence images were acquired under anesthesia at 1, 4, 24, 72 and 96 hrs. Animals were housed in subdued light conditions. Confocal fluorescence imaging (LSM 510 Meta (Zeiss, Germany)) was performed using a 40× objective. The distribution of Ce6 fluorescence was mapped under 514 nm laser excitation in the lambda mode (539–753 nm). White light transmission images were acquired to determine regions of interest (ROIs) within tumor, normal tissue and tissue vasculature. Spectra acquired from individual pixels were analyzed as a linear combination of basis spectra and fitted using a singular value decomposition algorithm. The fluorescence of Ce6 and conjugate **1** was determined in ROIs. No correction for tissue optical properties was performed. Three regions of interest in each chamber, for each tissue type, were recorded and weighted averages were used to determine pharmacokinetics. Values are presented as mean ± SE. Tests for significance between groups are performed using Student's t test where p<0.05 is deemed significant.

## Results

### Binding properties of conjugates

The sst_2_ mRNA expression levels in K562 WT and sst_2_+ cells are shown in Fig. 2A. Wt and K562 sst_2_+ cells did not express detectable levels of sst_1_, sst_3_ and sst_5_ (data not shown).

When tested each conjugate for its ability to compete with specific binding of [^125^I]-SS14 to membrane preparations of K562 sst_2_+, conjugate **1** was 40× less potent than unlabeled Octreotide (IC_50_ of 68 nM vs 1.7 nM, respectively). Conjugate **2** also competed with specific [^125^I]-SS14 binding, but with an almost 5× higher potency (14 nM) than conjugate **1**. Displacement curves are shown in Fig. 2B.

### Photophysical properties of conjugates

In PBS at pH = 7.4 the absorption maximas of both conjugates revealed the shift to longer wavelength compared to Ce6. The bathochromic shift of the Q band was most pronounced (659 nm for Ce6 in conjugate **1** and 661 nm in conjugate **2**, compared to 655 nm of un-conjugated Ce6) (Table 1). The maximum of Soret band was shifted from 402 nm for Ce6 alone, to 405 nm for conjugate **1** and **2**. Soret band broadening increased with the number of [Tyr3]-octreotate in the conjugate.

**Table 1:** Photophysical properties of Ce6, conjugate **1** and **2** in PBS.

Photophysical property	Ce6	Conjugate 1	Conjugate 2
Δ 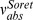 (nm) pH = 7.4	27	32	42
Δ 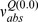 (nm) pH = 7.4	21	21	26
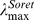 (nm) pH = 7.4	402	405	405
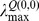 (nm) pH = 7.4	655	659	661
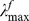 (nm) pH = 7.4	661	665	665
Φ_F_ in PBS pH = 7.4	0.18	0.20	0.03
k_blA_ (x 10^−2^ J^−1^ cm^2^)	25.69	14.07	22.70
Φ_Δ_ in PBS pH = 7.4	0.64±0.08	0.73±0.02	0.59±0.03
pKa	6.71±0.0947	5.38±0.0615	6.28±0.035
LogP pH = 7.4	0.005±0.05	0.679±0.04	2.820±0.34

Note: Δ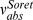 and Δ
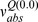
is the full width at half maximum of the Soret and Q(0,0) absorption band, respectively; 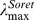 and 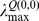 are the maxima of the Soret and Q(0,0) absorption bands, respectively; 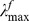 is the maximum of fluorescence band; LogP is the logarithm of partition coefficient; Φ_F_ is the fluorescence quantum yield and Φ_Δ_ is the singlet oxygen quantum yield, k_blA_ is rate constant of initial absorption bleaching and pKa represents the inflection point of pH titration curve (basis for titration curve was F_max_/A (λ_exc_)).

When the somatostatin analogue was conjugated with Ce6, the maximum of fluorescence intensity was red shifted (from 661 nm for Ce6 alone, to 665 nm for **1** and **2**). The fluorescence quantum yield of Ce6 in neutral pH was similar to conjugates **1** (Φ_F_ = 0.18 for Ce6 and Φ_F_ = 0.20 for **1**). The fluorescence quantum yield of conjugate **2** was significantly lower (Φ_F_ = 0.03).

**Figure 3:**
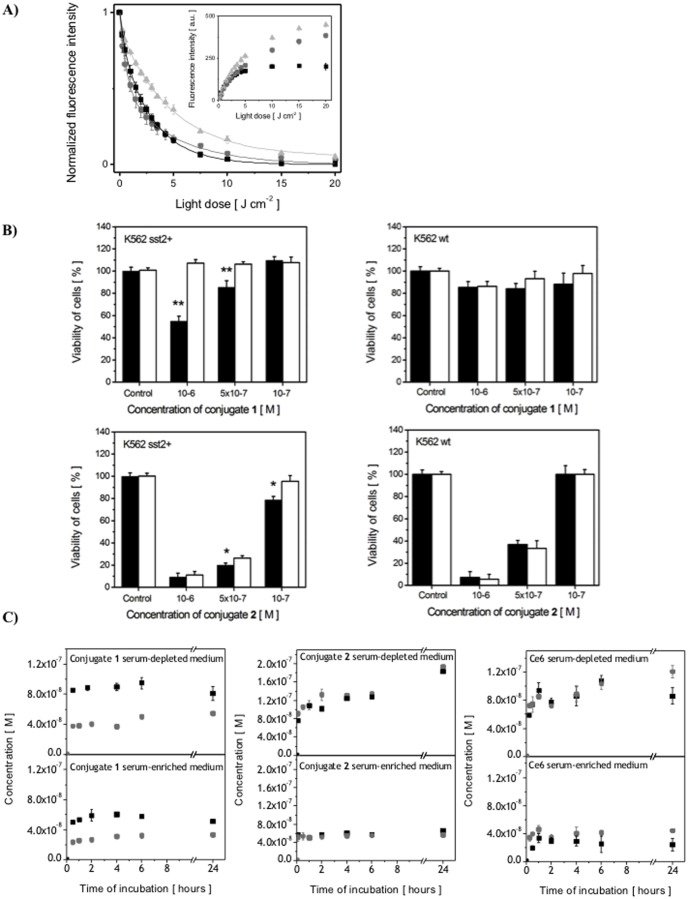
(A) Kinetics of fluorescence bleaching of photosensitizers (▪) Ce6, (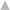) conjugate **1** and (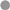) conjugate **2** in PBS under 652 nm irradiation (the fluorescence emission spectra of photosensitizers have been detected with the excitation wavelength λ_exc_ = 405 nm). Insert: Singlet oxygen (^1^O_2_) production by Ce6 and Ce6-conjugates; (B) Conjugates receptor-specificity proliferation of K562 sst_2_^+^ and WT cells after PDT (5 J.cm^−2^ light dose with 652 nm irradiation). Different concentrations of conjugates **1** and **2** were incubated with sst_2_^+^ and WT cells: (▪) in the absence of unlabeled Octreotide and (□) with Octreotide co-treatment (c = 10^−5^ M). Test of significance between the group of conjugates phototoxicity in absence and in presence of Octreotide has been performed using Student's test. In case of conjugate **1** value p<0.005 is deemed significant, whereas for conjugate **2** p<0.05; (C) The effect of the sst_2_ expression on the uptake of conjugate **1**, **2** and un-conjugated Ce6. The photosensitizers (c = 1 µM) were incubated with K562 cells in serum-free medium (top) and in the medium with 5% FCS (bottom): (▪) K562 sst_2_^+^, (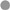) K562 WT cells.

The singlet oxygen production (Φ_Δ_) determined relative to Φ_Δ_ = 0.64 for Ce6 [37], was found Φ_Δ_ = 0.73 for conjugate **1** and Φ_Δ_ = 0.59 for conjugate **2**. Although the singlet oxygen production did not significantly differ between the compounds, un-conjugated Ce6 showed low photostability. During illumination above 5 J.cm^−2^ no fluorescence intensity was detected, without any further singlet oxygen production (Figure 3A). In contrast to un-conjugated Ce6, conjugates **1** and **2** revealed higher photostability. With higher irradiation dose (>5 J.cm^−2^), the conjugates continue to produce singlet oxygen (insert of Figure 3A).

### Effect of pH on properties of Ce6 and Ce6-conjugates

Compared to un-conjugated Ce6, the same spectral features were observed for both conjugates: the fluorescence emission maximum was blue shifted with decreasing the pH (Figures S2–S4 in File S1). The titration curves displayed inflection points at pH 6.71 for un-conjugated Ce6 (Figure S2 in File S1), at pH 5.38 for conjugate **1** (Figure S3 in File S1), and at pH 6.28 for conjugate **2** (Figure S4 in File S1).

### Partition coefficients

Octanol-water partition coefficient increased with the number of [Tyr3]-octreotate within the conjugate structure (Table 1). Conjugate **1** showed approximately 5× higher accumulation within the 1-octanol phase relative to Ce6, reflecting its higher hydrophobicity compared to Ce6 alone. In contrast, **2** had >600× higher accumulation in 1-octanol than in buffer and can be classified as hydrophobic.

### Phototoxicity

Irradiation of K562 sst_2_+ cells preincubated with conjugates caused phototoxicity in the concentration dependent manner (Figure 3B). While 1 µM concentration of conjugate **1** induced approximately 50% of cell survival compared to controls cells, with conjugate **2** only 10% of cell survived after 5 J.cm^−2^ (Figure 3B). Co-treatment of K562 sst_2_+ cells with unlabeled Octreotide (to prevent the binding of conjugates to sst_2_) abolished the inhibitory effect of both conjugates. However, the decrease in PDT toxicity after Octreotide co-treatment was more evident for conjugate **1**. In addition, while conjugate **1** did not show any toxicity in K562 WT cells (with or without Octreotide co-treatment), the PDT activity of conjugate **2** on K562 WT was comparable with the PDT activity on K562 sst_2_+ cells. Treatment of cells with the photosensitizer alone (Ce6 un-conjugated or conjugates), in the absence of light or light exposure alone, had no effect on cell growth.

### Intracellular Uptake

The role of the sst_2_ expression on cell uptake of the conjugates and un-conjugated Ce6 (1 µM) is shown in Figure 3C. Of the conjugates, only conjugate **1** showed specificity of uptake. An approximately 2× higher concentration was detected in sst_2_+ compared to WT cells. Enrichment of the medium with the serum decreased the uptake of conjugates by cells, but without any effect on the specificity of uptake. K562 sst_2_+ cells displayed 2× higher uptake of conjugate **1** compared to WT cells, in both serum-enriched and -depleted medium. In contrast no difference in concentration uptake of conjugate **2** between sst_2_+ and WT cells was detected. The same result as for conjugate **2** was observed also for un-conjugated Ce6.

### Comparison of Phototoxicity Induced by Conjugate 1 and Un-conjugated Ce6 on K562 Sst_2_+ Cell Line

**Figure 4:**
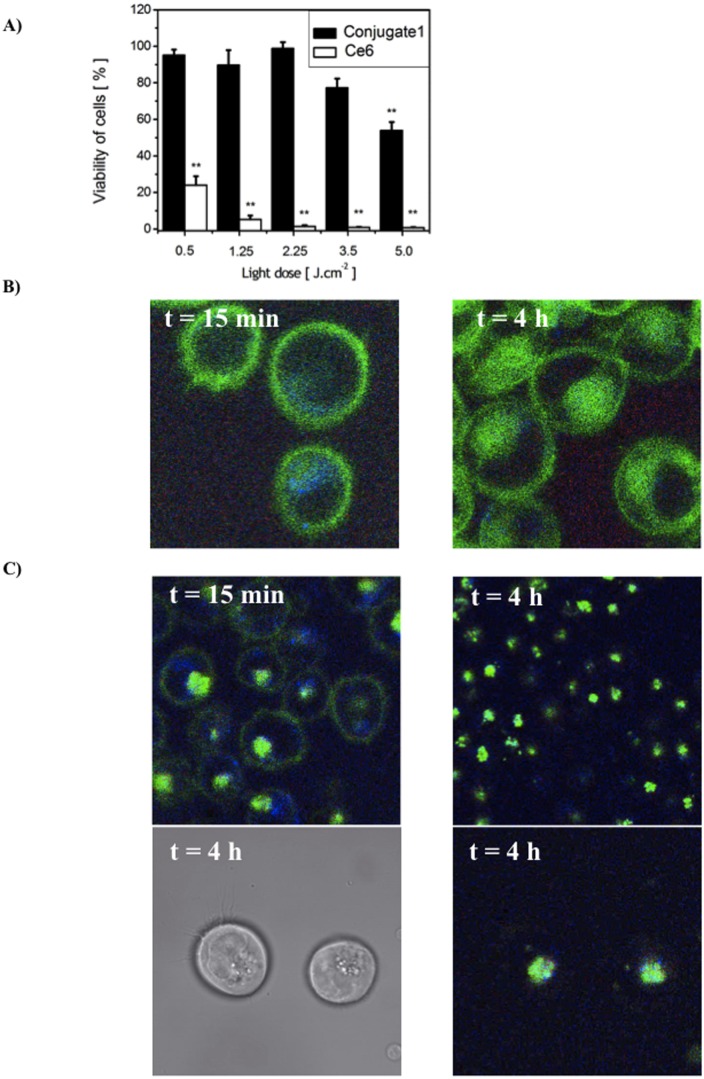
(A) PDT effects of un-conjugated Ce6 and conjugate **1** (c = 1 µM) on K562 sst_2_^+^ cell viability as a function of light dose. There is a statistically significant difference between the viability of cells incubated with un-conjugated Ce6 followed by irradiation with different light doses and the non-irradiated cells (groups of controls) (p<0.005). In case of conjugate **1**, only 5 J.cm^−2^ light dose shows the significant difference (p<0.005) when compare to its control (e.g. non-irradiated cells incubated with conjugate **1**); (B) Confocal fluorescence images of un-conjugated Ce6 in K562 sst_2_^+^ cells after 15 min and 4-h incubation in serum-free medium; (C) Confocal fluorescence images of conjugate **1** after 15 min (top left image) and 4-h incubation (top right image) in serum-free medium. Bottom right image shows conjugate **1** localization 4-h after incubation and its corresponding transmission image (bottom left).

The phototoxicity of K562 sst_2_+ cells attributed to the 4 h incubation with conjugate **1** and un-conjugated Ce6 and light exposure is shown in Figure 4A. Both photosensitizers induced a PDT effect in a light-dose dependent manner. However, for the same dose of light, the un-conjugated Ce6 was more potent than conjugate **1**. The un-conjugated Ce6 shows phototoxicity after 0.5 J.cm^−2^ with increased inhibition of cell growth with increasing light dose. This was in contrast to cells preincubated with conjugate **1**, for which the light dose dependent effect was seen from 3.5 J.cm^−2^. At 5 J.cm^−2^, Ce6 alone results in almost complete inhibition of cell viability, whereas cells preincubated with conjugate **1** showed a 50% cell survival.

### Intracellular Localization

The intracellular localization of un-conjugated Ce6 and conjugate **1** in K562 sst_2_+ cells is shown in Figures 4B and 4C. The comparison of cells treated with Ce6 alone and conjugate showed a clear difference in the distribution of the fluorescence. After 15 min incubation with Ce6 in serum-depleted medium, the fluorescence images revealed the presence of Ce6 in the plasma membrane. At 4 hours, strong staining of membrane structure together with perinuclear region was detected (Figure 4B). This was in contrast to the localization of conjugate **1** for which at 15 min the fluorescence was located in the outer membrane with some discrete localization pattern within the cytoplasm. The punctuate localization was maintained for longer incubation periods, with no evidence of fluorescence in the plasma membrane (Figure 4C).

### Fluorescence pharmacokinetics in subcutaneously implanted AR42J tumors

**Figure 5:**
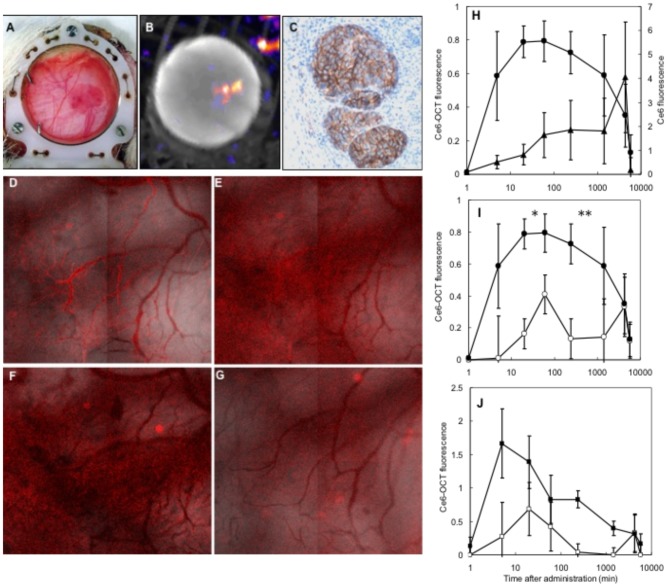
(A) AR42J tumor in the skin-fold observation chamber; (B) Corresponding OctreoScan-SPECT/CT fusion image; (C) Immunohistochemical sst_2_ stain; (D–G) Conjugate 1 fluorescence/white light transmission fusion images 5 min, 1 h, 4 h and 24 h after conjugate administration respectively; (H) In-vivo fluorescence pharmacokinetics of Ce6 (▴) (n = 3) and conjugate 1 (•) (n = 4) in AR42J tumor tissue in the rat skin-fold observation chamber; (I) In-vivo fluorescence pharmacokinetics of conjugate 1 in tumor tissue with (○) and without (•) the addition of free Octreotide; (J) In-vivo fluorescence pharmacokinetics of conjugate 1 in the tumor vasculature with (□) and without (▪) the addition of free Octreotide. There is a statistically significant difference between the fluorescence in tumor tissue between the blocked and the unblocked groups 20 min (p = 0.04) and 20 min (p = 0.004) after administration

Figure 5A–C shows a representative example of the AR42J in the rat chamber immediately prior to pharmacokinetic measurements. A white light image shows vital tumor tissue that is coincident with an OctreoScan-SPECT/CT fusion image showing uptake in sst_2_+ positive cells *in-vivo*. An immunohistochemical sst_2_+ stain shows the microscopic distribution of sst_2_+ cells surrounded by subcutaneous facia (normal tissue). The spatial distribution of pharmacokinetics from conjugate **1** is shown in Figure 5D–G. Five minutes after administration the conjugate **1** was predominately in the chamber vasculature. At 1 hour and in particularly 4 hours after administration increased fluorescence was observed in tumor tissue immediately surrounding the small vessels in the tumor microvasculature. After 24 hours conjugate **1** was clearing from tumor and normal tissue. Figure 5H–J shows the fluorescence pharmacokinetics of conjugate **1** in tumor tissue and in chamber vasculature. The pharmacokinetics of conjugate **1** in tumor tissue were significantly different from free Ce6. Conjugate **1** fluorescence peaks between 20 and 60 minutes whereas Ce6 gradually increased over the first 3 days after administration (Figure 5H). Both drugs were cleared during the time course of the experiment. Figure 5I shows that co-administration of Octreotide significantly reduced the uptake of conjugate **1** in tumor tissue. Figure 5J shows the uptake of conjugate **1** in tumor vasculature was also reduced when Octreotide was administered.

## Discussion

Tumor cells and tumor vasculature are both potential targets of PDT. However, the preference of cellular versus vascular targeting is dependent upon the relative distribution of photosensitizers in each compartment. This relative distribution is determined by the pharmacokinetics of the photosensitizer. In most cases effective PDT relies on the strategic choice of drug-light interval between the administration of photosensitizer and illumination [8]. In addition to the passive targeting approach, active targeting of tumor endothelial and cellular markers has been studied extensively [8], [9]. However, for targeted approaches, either only vasculature, or only tumor cells have been targeted. A high density of sst_2_^+^ has been reported in neuroendocrine tumors, angiogenic vessels and peritumoral vessels [17]–[21]. This makes sst_2_ an attractive target for tumor therapy, since it can target both tumor cells and tumor neovasculature simultaneously [12]–[21].

Our experiments showed that the conjugation of [Tyr3]-octreotate with the Ce6 improves its ability to target cancer cells over-expressing sst_2_. However, surprisingly, even though both conjugates showed receptor binding, specific in vitro targeting was only confirmed for one of the conjugates we studied. The decrease of phototoxicity of conjugate **1** on K562 sst_2_^+^ cells after Octreotide co-treatment together with no evidence of phototoxicity in K562 WT cells, can be explained by differences in concentration. A substantial increase in concentration of conjugate **1** by sst_2_^+^ cells compared to WT cells illustrates the role of sst_2_ in the uptake of conjugate **1**. This was in contrast to conjugate **2** where although conjugate **2** was found to be a more potent photosensitizer, no difference in the intracellular concentration of conjugate **2** was demonstrated in sst_2_^+^ compare to WT cells. Similarly, its PDT effect on sst_2_^+^ cells was not inhibited by Octreotide co-treatment and showed comparable phototoxicity in WT cells. These results indicate the absence or a minor role of specific uptake of conjugate **2** by sst_2_. This result is in contrast to the binding affinity of conjugate **2** which was found to have a high affinity to sst_2_. This discrepancy is likely to be a consequence of the fact that conjugate **2** is highly hydrophobic. Because of that the nonspecific accumulation within the cell dominated the specific recognition of conjugate **2** by sst_2_.

A number of authors have used Ce6 for conjugation with either tumor specific monoclonal antibodies [4]–[7], or in peptide mediated approaches with insulin [41] or transferrin [42]. Akhlynina et al. [41] demonstrated superior phototoxicity of conjugates compared to un-conjugated Ce6. However, as the authors stated, despite promising results, the direct applicability in cancer therapy is limited, due to the low expression of insulin receptors on most hepatoma cells. This shows that while conjugation enhanced phototoxic properties of conjugate compared to free Ce6, the selectivity of conjugate uptake can be hampered by low receptor expression.

In the work of Del Governatore [5], several Ce6 molecules were covalently attached to anti-colon cancer monoclonal antibody 17.1A, in a way that conjugates had either cationic or anionic charges. Both immunoconjugates showed in vitro selectivity for antigen-positive target cells with a specificity of uptake (by a factor of approximately 1.9) that is similar to what we have found in the present study. Further in vivo biodistribution studies by the same investigators [4] showed that the anionic immunoconjugate showed both a higher absolute uptake of Ce6 in tumor tissue and a superior selectivity for tumor over normal tissue.

It is interesting to consider our finding that conjugate **1** is the least efficient photosensitizer at equivalent concentration of incubation and light fluence. There are a number of potential explanations for the higher efficacy of un-conjugated Ce6 than of conjugate **1**. First, the quantitative uptake of Ce6 into cells may play an important role in efficacy. Second, as a result of conjugation, the photochemical reactivity of un-conjugated Ce6 versus conjugate might differ and third, the site of intracellular localization is different of the photosensitizer and is known to be an important factor [10]. Our concentration uptake data showed that for a 4 h incubation period, un-conjugated Ce6 and conjugate **1** are taken up by sst_2_^+^ cells equally, (9×10^−8^ M was found for both photosensitizers). This means, that the higher PDT activity of Ce6 on sst_2_^+^ cells cannot be related to uptake differences.

To investigate our second hypothesis, the photophysical properties of each photosensitizer(-conjugate) were determined. The spectroscopic changes for both conjugates and these were most significant for conjugate **2** (broadening of the absorption spectra and decrease in fluorescence quantum yield as a result of increase hydrophobicity and insolubility in aqueous media). The quantum yield of ^1^O_2_ observed with un-conjugated Ce6 was not significantly altered by conjugation of [Tyr3]-octreotate molecule to Ce6. In fact, ^1^O_2_ production was higher for conjugate **1** (Φ_Δ_ = 0.73±0.02) than that for Ce6 (Φ_Δ_ = 0.64±0.08). Figure 3 also illustrates that conjugate **1** is more photo-stable. This might be explained by the involvement of peptide in the bleaching process. Although conjugate **1** has a high quantum yield of ^1^O_2_ production, the presence of peptide seems to prevent the attack of the tetrapyrrole ring. It is known that the presence of aminoacids, in this case within the conjugate, can influence the consumption of reactive oxygen species [43], [44]. However, the mechanisms underlying these processes are likely to be complex. This is illustrated by the fact that conjugate **2** showed a similar rate of photobleaching to Ce6. Here the spatial conformation of the amino acids and thus their susceptibility to ^1^O_2_ attack may be different. Clearly, while additional information about the peptide conformation within conjugates **1** and **2** is required to fully understand these kinetics of photobleaching, our data show that differences in concentration, photostability and ^1^O_2_ generation do not explain the higher phototoxicity of un-conjugated Ce6 compared to conjugate **1**.

Therefore, of the three hypotheses, the later, a localization difference would appear to play the dominant role. Upon irradiation, cytotoxic products will be formed at sites where photosensitizers are localized. Photosensitizers initially located in lysosomes, kill cells less efficiently than those targeted at the mitochondria or endoplasmatic reticulum [45]. In our study, we showed a dramatically different localization pattern for un-conjugated Ce6 compared to conjugate **1**. Fluorescence of un-conjugated Ce6 was present diffusely in cellular membranes. In contrast, conjugate **1** was sequestered in discrete, intracytoplasmic compartments. Since conjugate **1** is specifically recognized by the sst_2_ receptor it is likely that the sst_2_ endocytosis pathway [12], [13] is the main mechanism of the internalization of conjugate **1**.

Moreover, the highly localized pattern of conjugate accumulation for all of the incubation times shows that conjugate **1** does not diffuse into other cell compartments. Our data on the spectral properties of un-conjugated Ce6 and Ce6-conjugates shows evidence of pH-dependent modifications of photosensitizers in the physiological relevant pH range. The inflection point at pH 5.38±0.06 of conjugate **1** is well within pH range associated with the somatostatin receptor endocytosis pathway. A pH as low as 4.7 has been determined during this vesicular maturation [46]. Changes in the ionization state of molecule might mean it is difficult for the conjugate to pass the membrane of the endosomal/lysosomal compartments [47] and thus be trapped within these organelles. High local concentrations of photosensitizers and their conjugates are known to lead to the formation of aggregates that have reduced PDT activity [48]. While the formation of aggregates in endosomal compartments may represent a significant limitation of our approach an interesting future approach may be the design of novel light treatment regimens that utilize fractionated illumination to induce relocalization of the conjugate from its original localization site and/or its disaggregation during dark intervals in therapy.

Given the differences in *in-vitro* results for each conjugate, conjugate **1** was selected to determine *in-vivo* pharmacokinetics in an sst_2_^+^ tumor model. Encouragingly the pharmacokinetics of fluorescence in AR42J tumors showed conjugate **1** exhibited a receptor mediated uptake that can be abrogated with co-administration of Octreotide. The magnitude of this reduction in fluorescence with co-treatment with Octreotide is a useful indicator of the *in-vivo* selectivity of conjugate **1** in sst_2_^+^ tumor tissue. Between 20 minutes and 4 hours after the administration of conjugate **1** there was on average >4× less fluorescence after the administration of Octreotide. This level of selectivity is somewhat larger than that we found in our *in-vitro* studies. This method of assessing selectivity has the advantage that the same type of tissue is being compared and therefore overcomes differences in tissue optical properties (blood absorption and differences in scattering) that are known to influence fluorescence imaging techniques. It is also important to recognize that Octreotide is not present in excess since the effective concentration was lower than that of conjugate **1**. This may lead to a small underestimate of the selectivity of conjugate **1** for sst_2_^+^ tumor tissue. It is also encouraging that we observe at least a partial sst_2_ receptor mediated uptake in the tumor vasculature that was also reduced by co-administration with Octreotide. This result illustrates the potential of a dual strategy targeting sst_2_^+^ tumour and neo-vasculature that is known to also express the sst_2_ receptor. While the rat-skin fold window chamber transplanted with sst_2_^+^ AR42J acinar pancreas tumors was used to determine the pharmacokinetics of conjugate **1**, we believe that this model is not suitable to perform PDT toxicity studies, since the distribution of oxygen and light are important factors. In a previous study we have shown, that it is possible to simultaneously monitor the photosensitizer photobleaching, in addition to fluence (rate), hemoglobin oxygen saturation, and blood volume during PDT, without interruptions to the therapeutic illumination [49]. Since these parameters are important factors that influence the outcome of PDT, we aim to incorporate these measurements in future therapeutic studies *in-vivo* in orthotopic tumor models.

We are not the first to investigate the use of sst targeted PDT *in-vivo*. A group from Montana State University has previously reported results in which tetrapyrrole based photosensitisers are conjugated with octreotate-targeting peptides that are being studied for the application of multi-photon activated PDT [50], [51]. While the results of these pre-clinical studies are encouraging it should be noted that they have been achieved using the local infiltration of photosensitizer conjugate. This administration route was chosen because therapeutic doses could not be injected i.v. via the mouse tail vein because of the high viscosity of their photosensitizer conjugate. In the present study we have chosen to administer conjugates systemically using an i.v injection because we believe that this administration route has a significantly greater translational potential.

In summary, our studies have clearly shown the impact of conjugation on photosensitizer properties. The differences between our conjugates demonstrate the necessity of careful study of the photochemical and photophysical properties of photosensitizer, when the conjugation is involved. This knowledge is important not only as a potential predictor of success of a conjugate as a PDT agent but as we have shown, the physicochemical properties of conjugates are important factors in the process of internalization and specificity of cellular uptake. While these initial *in-vitro* and *in-vivo* data are encouraging, there remain questions, which will only be answered by PDT response studies *in-vivo*.

## Supporting Information

File S1 Synthesis of Ce6-coupled somatostatin analogues and characterization.
(DOC)
