# Supplementary material for: Somatostatin Analogues for Receptor Targeted Photodynamic Therapy
Source: PLoS One. 2014 Aug 11;9(8):e104448. doi: 10.1371/journal.pone.0104448 (PMC4128677; doi:10.1371/journal.pone.0104448)
Supplement: File S1 — Synthesis of Ce6-coupled somatostatin analogues and characterization. (DOC) [file pone.0104448.s001.doc]

**Supporting Information**

**Synthesis of Ce6-coupled somatostatin analogues and characterization.**

**Solvents and Reagents.** N-hydroxybenzotriazole (HOBt) was purchased from AnaSpec (San Jose, CA). Trifluoroacetic acid (TFA) and N, N-diisopropylethylamine (DIEA) were purchased from Advanced ChemTech (Louisville, KY). Dichloromethane (DCM), *N, N*-dimethyformamide (DMF), methanol, and acetonitrile were from purchased from Fisher Scientific. Ce6 was purchased from Frontier Scientific, Inc. (Logan, Utah). Diisopropylcarbodiimide (DIC) and other commercial chemicals were purchased from Sigma-Aldrich. All solvents and chemicals were used without further purification.

**Synthesis.** As summarized in Scheme S1, the linear protected peptide, Fmoc-DPhe-Cys(Acm)-Tyr(But)-DTrp(Boc)-Lys(Boc)-Thr(But)-Cys(Acm)-Thr(But), was assembled from Fmoc-Thr(But)-Wang resin using Fmoc chemistry. The disulfide bond was formed with thallium trifluoroacetate in DMF on solid support. After the three Lys residues were introduced similarly using Fmoc chemistry, Ce6 was conjugated at the N-terminus of the peptide in the presence of HOBT and DIC in DMF. Finally, the desired product, Ce_6_-K_3_-[Tyr3]-octreotate, was obtained by TFA-mediated cleavage and HPLC purification.

As summarized in Scheme S2, the resin-bound protecting [Tyr3]-octreotate was further elongated with Fmoc-DPhe-Cys(Acm)-Tyr(But)-DTrp(Boc)-Lys(Boc)-Thr(But)-Cys(Acm)-Thr(But) via Fmoc chemistry, followed by oxidation with thallium trifluoroacetate in DMF on the solid support. Similarly, Ce6 conjugation, TFA-mediated cleavage, and HPLC purification afforded the dimeric compound, Ce_6_-[Tyr3]-octreotate-K_3_-[Tyr3]-octreotate.

**Solid phase peptide synthesis.** Solid phase peptide synthesis was performed as follows: All peptides were assembled manually on resin using conventional Fmoc chemistry. The coupling reactions were carried out by adding a pre-activated solution of N-α-Fmoc-protected amino acid (3 equiv), 1-hydroxybenzotriazole (HOBt, 3 equiv), 2-(1H-benzotriazole-1-yl)-1,1,3,3-tetramethyluronium hexafluorophosphate (HBTU, 3 equiv), and N,N-diisopropylethylamine (DIEA, 6 equiv) in anhydrous N,N-dimethylformamide (DMF, 3 mL/g resin) to the resin (1 equiv) and mixing for 2 h. The progress of the coupling was monitored by the Kaiser test. The Fmoc protecting group was removed with a solution of 20% piperidine in DMF for 10 min (2X). The resin was washed with methanol (1 min, 3 mL, 2X) and DMF (1 min, 3 mL, 6X) and used in subsequent reactions.

**Fmoc-protected-[Tyr3]-octreotate-Wang resin.** The resin-bound protected peptide Fmoc-DPhe-Cys(Acm)-Tyr(But)-DTrp(Boc)-Lys(Boc)-Thr(But)-Cys(Acm)-Thr(But)-Wang resin was assembled manually from Fmoc-Thr(But)-Wang resin (1.0 g, ~0.20 mmol/g) as described above. Thallium trifluoroacetate (1 equiv) in DMF (5 mL) was added and the mixture was swirled for 1.5 h. This step was repeated for another time. The resin was washed with DMF (3X) and methanol (2X). Finally, the resin was dried and divided into 4 portions for the following reactions.

**Ce6-K_3_-[Tyr_3_]-octreotate (conjugate 1):** One portion of the resin obtained above (~0.05 mmol) was used in synthesis of Fmoc-Lys3-protected [Tyr3]-octreotate-Wang resin. Fmoc-Lys3-protected-[Tyr3]-octreotate-Wang resin was deblocked with piperidine in DMF (20%), washed with methanol (2X) and DMF (5X). A mixture of Ce6 (3 equiv), HOBT (3 equiv), and DIC (3 equiv) in DMF was added into the resin and swirled overnight. The mixture was filtrated and washed with DMF (2X) and methanol (3X). The resin obtained was cleaved with TFA/water/thioanisole/phenol/EDT (82.5:5:5:5:2.5, 3 mL) for 1 min (3X). The filtrate was concentrated to give the crude product, which was purified by semi-preparative HPLC. After identified by both ES-MS and analytical HPLC, the desired fractions were combined and lyophilized to get the title compound (15mg, ~13% yield). ES-MS observed: [MH]2+/[MH]^3+^ 1007.15/671.60). Some other peaks deduced to be the corresponding species from K^+^ and Na^+^ complexation.

**Ce6-[Tyr_3_]-octreotate-K_3_-[Tyr_3_]-octreotate (conjugate 2):** Similarly, one portion Fmoc-protected [Tyr3]-octreotate-Wang resin (~ 0.05 mmol) was used in synthesis of this title compound. The resin-bound peptide was further elongated with Fmoc-DPhe-Cys(Acm)-Tyr(But)-DTrp(Boc)-Lys(Boc)-Thr(But)-Cys(Acm)-Thr(But) via Fmoc chemistry. Thallium trifluoroacetate (2 equiv) in DMF was added and swirled for 2 h. The resin was washed and deblocked with 20% piperidine in DMF. Ce6 was conjugated using a mixture of Ce6 (3 equiv), HOBT (3 equiv), and DIC (3 equiv) in DMF as described above. TFA-mediated cleavage (TFA/water/thioanisole/phenol/EDT: 82.5:5:5:5:2.5, 3 mL) and HPLC purification afforded the title compound (7mg, ~4% yield). ES-MS observed: [MH]3+/[MH]4+/[MH]5+/[MH]^7+^ 1015.25/761.60/609.75/437. Some other peaks deduced from related K^+^ and Na^+^ complexations were also observed.

**Purification and Analysis.** HPLC analysis was performed on a Phenomenex C-18 column (Jupiter 5u, 5 micron, 300A, 250x4.6 mm) at a flow rate of 1.0 mL/min. Semi-preparative HPLC was performed on a Vydac C-18 column (25X2.2 cm) at 9.5 mL/min. HPLC solvents consist of water containing 0.05% TFA (solvent A) and acetonitrile containing 0.05% TFA (solvent B). The elution protocol for analytical HPLC starts with 90% A for 3 min, followed by a linear gradient to 30% A over 6 min, continuing to 50% A over 14 min and to 10% A over 2 min, held at 10% A for 3 min, and finally returning to 90% A over 1 min. The elution profile was monitored by UV absorbance at 254 nm and 214 nm. Mass spectra were obtained using Shimadzu mass spectrometer (LCMS-2010A) in the positive electro-spray mode. Figure S1 shows the ES-MS and HPLC profiles of conjugates **1** and **2**.

**Partition coefficient.** Stock solutions of Ce6 and conjugates (0.6 mM) were prepared in DMSO. Lipophilicity was characterized by their partition coefficient P_oct/buffer_ = C_oct/_C_buffer_ between two solvents 1-octanol (oct) and PBS, pH = 7.4 (buffer). Measurements were carried out in duplicates as described by Cunderlikova et al [23].

**Steady-State Fluorescence and Absorption Measurements.** The fluorescence quantum yields (Φ_F_) of Ce6 and conjugates in PBS pH 7.4 were determined by a relative method [31]. Ce6 in the buffer at pH 7.4 with the quantum yield of 0.18 [24,32,33] was used as a reference.

The absorption profile of Ce6 and conjugate in PBS of concentration (0.06–1μM) was recorded in Shimadzu spectrophotometer (UV-2101 PC, Duisburg, Germany).

The fluorescence emission of photosensitizers was recorded in a Perkin-Elmer spectrofluorimeter (LS 50B, Massachussetts, USA). Light at 408 nm was used for excitation. The concentration range of compounds matched to the O.D. up to 0.1.

**Photobleaching measurements.** The loss of photosensitizer absorbance and fluorescence during irradiation was used to monitor photobleaching. Solutions of Ce6 and Ce6-conjugates were prepared in PBS pH = 7.4. The concentration of photosensitizers were adjusted so that all absorb the same number of photons at irradiated region (O.D. = 0.014 at the λ_irr_. = 652 nm (Biolitec pharma, UK). Data was acquired in quartz cuvettes in aerobic conditions using stirring under uniform, measured irradiation [34] at 5 mWcm^-2^. At intervals, the cuvette was removed from magnetic stirrer and absorption and fluorescence emission spectrum (λ_exc_ = 405 nm) acquired. The initial absorption bleaching rate constants (k_blA_) for Ce6 and conjugates were evaluated as reported [27].

**Singlet Oxygen Quantum Yield Measurements.** Photooxidation of Singlet Oxygen Sensor Green Reagent (SOSGR) (Molecular Probes, NL) was used to determine the singlet oxygen formation by Ce6 and Ce6-conjugates. We used SOSGR because of its high selectivity for singlet oxygen and low sensitivity to hydroxyl radicals/superoxides [35]. Stock solution of SOSGR (5 mM) was prepared in methanol. The final concentration of SOSGR within the photosensitizer solution in PBS pH 7.4 was 1 μM. The irradiation was performed the same as for photobleaching measurements described above. The singlet oxygen quantum yields (Φ_△_) of conjugates in PBS were determined in duplicate by a relative method [36] with a Φ_△_ = 0.64 of Ce6 in the PBS at pH 7.4 as reference [37].

**pH measurements.** The pH titration of PBS was performed with HNO_3_ and NaOH over pH 4.0-8.5. The final concentration of Ce6 and Ce6-conjugates in solution was 0.8 μM. The pH was measured by Sentron pH meter (Sentron Europe, NL) and the absorption and fluorescence emission (λ_exc_ = 408 nm) profiles of solutions were recorded. The inflection point of titration curves (pKa) were evaluated as reported [23]. Briefly, the results of fluorescence titrations are presented in terms of a plot of fluorescence intensity as a function of pH with the constant concentration of the compound used throughout measurements. However, variations of fluorescence intensity are due to at least two contributions: the changes of molar extinction coefficient (differed absorbed intensity) and the changes of quantum efficiency with pH. To eliminate the influence of the molar extinction coefficient variations, the ratio of fluorescence intensity at the peak position to the absorbance at the excitation wavelength was plotted, followed by sigmoidal function fitting procedure (Figures S2-S4).

**Suporting Information Legends**

**Scheme S1**

Synthesis of Ce_6_-K_3_-[Tyr3]-octreotate (conjugate **1**)**:** The linear protected peptide was assembled from Fmoc-Thr(But)-Wang resin using Fmoc chemistry. The disulfide bond was formed with thallium trifluoroacetate in DMF on solid support. Ce6 was conjugated at the N-terminus of the peptide i.e. Lys3-[Tyr3]-octreotate. Finally, the desired product was obtained by cleavage with TFA/water/thioanisole/phenol/EDT (82.5:5:5:5:2.5) and HPLC purification.

**Scheme S2**

Synthesis of Ce_6_-[Tyr3]-octreotate-K_3_-[Tyr3]-octreotate (conjugate **2**): The resin-bound protecting [Tyr3]-octreotate peptide obtained from scheme 1 was further elongated via Fmoc chemistry, followed by the second disulfide bond formation and Ce6 conjugation. Finally, TFA cleavage and HPLC purification afforded the desired dimeric compound.

**Figure S1**

The ES-MS and HPLC profiles of A) conjugate **1** and B) conjugate **2**.

**Figure S2**

Effect of pH on A) fluorescence emission spectra (λ_exc_ = 408 nm) of Ce6 (c = 0.8 μM) in PBS. B) Ratio of fluorescence intensity at the peak position to the absorbance at the excitation wavelength as a function of pH for Ce6 in PBS.

**Figure S3**

Effect of pH on A) fluorescence emission spectra (λ_exc_ = 408 nm) of conjugate **1** (c = 0.8 μM) in PBS. B) Ratio of fluorescence intensity at the peak position to the absorbance at the excitation wavelength as a function of pH for conjugate **1** in PBS.

**Figure S4**

Effect of pH on A) fluorescence emission spectra (λ_exc_ = 408 nm) of conjugate **2** (c = 0.8 μM) in PBS. B) Ratio of fluorescence intensity at the peak position to the absorbance at the excitation wavelength as a function of pH for conjugate **2** in PBS.

**Scheme S1**

**Scheme S2**

**Figure S1**

**A)**

Minutes

2

4

6

8

10

12

14

16

18

20

22

mAU

0

500

1000

mAU

0

500

1000

**UV Ch2-220nm**

**B)**

Minutes

2

4

6

8

10

12

14

16

18

20

22

24

26

mAU

0

200

400

mAU

0

200

400

**UV Ch2-220nm**

**Figure S2**

**A)**

**B)**

**Figure S3**

**A)**

**B)**

**Figure S4**

**A)**

**B)**
